# Marvels of Bacilli in soil amendment for plant-growth promotion toward sustainable development having futuristic socio-economic implications

**DOI:** 10.3389/fmicb.2023.1293302

**Published:** 2023-12-07

**Authors:** Meenakshi Mukhopadhyay, Ashutosh Mukherjee, Sayak Ganguli, Archisman Chakraborti, Samrat Roy, Sudeshna Shyam Choudhury, Vetriselvan Subramaniyan, Vinoth Kumarasamy, Amany A. Sayed, Fatma M. El-Demerdash, Mikhlid H. Almutairi, Anca Şuţan, Bikram Dhara, Arup Kumar Mitra

**Affiliations:** ^1^Department of Botany, Vivekananda College (Affiliated to University of Calcutta), Kolkata, West Bengal, India; ^2^Department of Biotechnology, St. Xavier’s College (Autonomous), Kolkata, West Bengal, India; ^3^Department of Physics, St. Xavier’s College (Autonomous), Kolkata, West Bengal, India; ^4^Depatrment of Commerce, St. Xavier’s College (Autonomous), Kolkata, West Bengal, India; ^5^Post Graduate Department of Microbiology, St. Xavier’s College (Autonomous), Kolkata, West Bengal, India; ^6^Pharmacology Unit, Jeffrey Cheah School of Medicine and Health Sciences, Monash University, Bandar Sunway, Malaysia; ^7^Center for Transdisciplinary Research, Department of Pharmacology, Saveetha Dental College, Saveetha Institute of Medical and Technical Sciences, Saveetha University, Chennai, Tamil Nadu, India; ^8^Department of Parasitology and Medical Entomology, Faculty of Medicine, Universiti Kebangsaan Malaysia, Kuala Lumpur, Malaysia; ^9^Zoology Department, Faculty of Science, Cairo University, Giza, Egypt; ^10^Department of Environmental Studies, Institute of Graduate Studies and Research, Alexandria University, Alexandria, Egypt; ^11^Zoology Department, College of Science, King Saud University, Riyadh, Saudi Arabia; ^12^Department of Natural Sciences, Faculty of Science, Physical Education and Informatics, University of Pitești, Pitești, Romania; ^13^Center for Global Health Research, Saveetha Medical College and Hospital, Saveetha Institute of Medical and Technical Sciences, Chennai, Tamil Nadu, India

**Keywords:** sustainable agriculture, novel consortium of *Bacillus zhangzhouensis*, *B. subtilis*, *B. cereus*, multi-strain PGP bacterial inoculant, microbe-assisted bioaugmentation, plant-growth enhancement, resident bacterial community modulation

## Abstract

Microorganisms are integral components of ecosystems, exerting profound impacts on various facets of human life. The recent United Nations General Assembly (UNGA) Science Summit emphasized the critical importance of comprehending the microbial world to address global challenges, aligning with the United Nations Sustainable Development Goals (SDGs). In agriculture, microbes are pivotal contributors to food production, sustainable energy, and environmental bioremediation. However, decades of agricultural intensification have boosted crop yields at the expense of soil health and microbial diversity, jeopardizing global food security. To address this issue, a study in West Bengal, India, explored the potential of a novel multi-strain consortium of plant growth promoting (PGP) *Bacillus* spp. for soil bioaugmentation. These strains were sourced from the soil’s native microbial flora, offering a sustainable approach. In this work, a composite inoculum of *Bacillus zhangzhouensis* MMAM, *Bacillus cereus* MMAM3), and *Bacillus subtilis* MMAM2 were introduced into an over-exploited agricultural soil and implications on the improvement of vegetative growth and yield related traits of Gylcine max (L) Meril. plants were evaluated, growing them as model plant, in pot trial condition. The study’s findings demonstrated significant improvements in plant growth and soil microbial diversity when using the bacterial consortium in conjunction with vermicompost. Metagenomic analyses revealed increased abundance of many functional genera and metabolic pathways in consortium-inoculated soil, indicating enhanced soil biological health. This innovative bioaugmentation strategy to upgrade the over-used agricultural soil through introduction of residual PGP bacterial members as consortia, presents a promising path forward for sustainable agriculture. The rejuvenated patches of over-used land can be used by the small and marginal farmers for cultivation of resilient crops like soybean. Recognizing the significance of multi-strain PGP bacterial consortia as potential bioinoculants, such technology can bolster food security, enhance agricultural productivity, and mitigate the adverse effects of past agricultural activities.

## 1 Introduction

Intensification of crop production through extensive use of agrochemicals has been the major driving force for a quantum leap in crop yield after the green revolution. Over-exploitation of arable lands to feed the rapidly growing world population has negatively impacted the structure and function of soil by depleting nutrient levels, lowering microbiological diversity, and crop productivity (Huang et al., 2019) posing a serious threat to global food security. The damaged soils fail to regain their fertility satisfactorily and are unable to regenerate naturally (Goenster et al., 2017). Soils contain a myriad of microorganisms which constitute a characteristic microbiome playing a pivotal role in functioning of ecosystem and thereby, maintaining soil health (Maron et al., 2018). Research works spanning over the last few decades have established the importance of beneficial soil microbes, which can directly influence plant growth through mobilization of soil nutrients, secretion of plant beneficial secondary metabolites (such as phytohormones, siderophore) as well as indirectly protect plants from biotic and abiotic stresses, producing various stress metabolites (Bhattacharyya and Jha, 2012; Gouda et al., 2018).

In the rapidly growing sectors of sustainable agriculture, beneficial soil microorganisms (BSM) are assumed to steer the bio-based revolution in the near future as a potential alternative to complement or replace chemical fertilizers and pesticides (Malusà et al., 2021; Manfredini et al., 2021). Soil microbiome based approach has emerged as a promising strategy to mitigate the problem of soil productivity depletion, in an eco-friendly way (O’Callaghan et al., 2022). A healthy microbiome can be established in the crop field through the introduction of plant growth-promoting microbes (PGM) which exert their plant-favorable activity after building up a critical amount of biomass into the soil (Vassileva et al., 2020). These modulations may alter plant performance and soil health, and thereby, inducing unpredictable feedback reactions (Berg et al., 2021). It is well established that PGM having high enzymatic activity, phytohormone, and osmolytic metabolite-producing potential are effectively involved in plant health promotion and nutrient mobilization (Haas and Keel, 2003; Shilev et al., 2019). A recent study conducted by Kumar et al. (2023) on wheat rhizosphere in Upper-Gangetic plain, showed that various soil factors and conditions (e.g. available nitrogen, potassium, organic carbon) can be effectively improved through appropriate microbiome manipulation strategies.

Furthermore, some natural products (like vermicompost, agro-industrial wastes, etc.) can contribute to improving microbial diversity and promotes the growth of indigenous soil microbes within the soil–plant system (Arancon et al., 2006; Strachel et al., 2017). Implementation of organic farming through successful utilization of PGP microbes, protects and preserves soil health through sustainable and eco-friendly crop management practice by conservation and restoration activities (Gamage et al., 2023). Most of the studies in this field have revolved around utilization of single-strain microbial inoculants having multifarious plant growth promoting (PGP) activity. A recent study Hu et al. (2021) suggested a unique strategy of designing inoculants to rejuvenate the resident beneficial soil microbes (BSM) already present in the rhizosphere, using multi-strain microbial inoculants having multiple PGP traits. The effect of the introduced microbial inoculants may be transient or prolonged in the soil and it is dependent on the diversity of autochthonous soil microbial communities (Mallon et al., 2015; Mawarda et al., 2020). However, the potential of multi-strain residual bacterial consortia as an effective microbial inoculant to boost up crop production in long-term used nutrient depleted agricultural soil, is yet to be properly explored, especially in West Bengal, India. In this context, the objective of the present investigation was to explore the implications of soil amendment with a novel consortium of three residual PGP *Bacillus* spp., pre-isolated from the resident microbial flora of an over-exploited land. Vermicompost was used as a natural additive along with the multi-strain bacterial inoculant during soil augmentation. The soil collected from the same sampling field from where PGP *Bacillus* spp. were isolated, was used to carry out the whole study. Implications of this soil augmentation practice on the improvement of vegetative growth and yield related characteristics of *Gylcine max* (L) Meril. plants were evaluated, growing them as test plant, in pot trial condition. Furthermore, the potentiality of the present strategy to improve the resident bacterial community health of the nutrient-depleted soil, was also investigated.

## 2 Materials and methodology

### 2.1 Sampling site and collection of soil sample

The collection of soil samples was conducted from a nutrient-depleted arable land located at Bahadurpur, South 24 Parganas District, West Bengal, India, (21^0^ 26’ N-22^0^ to 38’ N, 87° 57′ to E-89° 09′E), abiding by the protocol of TNAU (2013).^1^ The soil was fine-loamy, Aeric-Epiaquerts type (Khan and Kar, 2020). Soil nutrient status like, organic (SOC) carbon, and available nitrogen (N), phosphorus (P), and potassium (K) content was estimated using standard methods as followed by Jackson (1967) and Mandal et al. (2020). For metagenomic analysis, fresh soil samples from the field and pots of each experimental set-up (at the fruit harvesting stage of soybean plants) were collected and pooled separately. Collected soil samples were stored separately at-20°C refrigerator until DNA extraction. For conducting pot experiments freshly procured non-sterilized soil from the sampling field was used.

### 2.2 Selection of PGPB strains and bacterial inoculant formulation

Three potent pre-isolated plant-growth promoting bacteria (PGPB) strains, identified by 16S rRNA sequencing as *Bacillus zhangzhouensis* MMAM (Accession no. MT 185655), *Bacillus cereus* MMAM3 (Accession no. MT 730003), and *Bacillus subtilis* MMAM2 (Accession no. MT 72561.1), were used as a novel consortium in this work. They were isolated from the resident microbial flora of the over-exploited soil, used in this investigation, and reported to have N-fixing, P-solubilizing, phytohormone Indole acetic acid (IAA), Gibberellic acid (GA) producing, and siderophore secreting ability. Additionally, these isolates can produce antimicrobial metabolites such as, amylase, protease, catalase, peroxidase, ammonia, and hydrogen cyanide (Mukhopadhyay, 2022). Fresh broth cultures of each of the isolates were prepared separately (HiMedia) incubating them in inoculated Luria Bertani (LB) (HiMedia) media for 48 h(h) at 37^o^ C in an orbital shaker at 160 rpm. The cell counts of the freshly grown bacterial suspensions were adjusted to 4.5×10 mL^−1^ (per milliliter). The microbial consortium suspension contained each of the three individual bacterial cultures at a 1:1:1 ratio. The inoculant formulation constituted 20% of the broth culture of the consortium, 30% sterilized distilled water, 30% sunflower oil, and 20% Tween-80 (Sigma. P6224).

### 2.3 Evaluation of *in vivo* growth-promotion efficacy of the bacterial consortium

The pot trial experiment was designed to assess *in vivo* plant growth promotion ability of the amendments in controlled conditions. *Glycine max* Meril. var. JS-0335 was used as the test plant. The seeds were procured from the ICAR-Indian Institute of Soybean Research, Indore. The non-sterilized, freshly collected soil from the field was used for this work. Seeds were surface sterilized using sodium hypochlorite solution (0.1%) for 5 min and were then rinsed thoroughly with sterile distilled water for 5 times before sowing in pots.

#### 2.3.1 Experimental design

The pot trial experiment was carried out in polythene bags of 28 centimeter (cm) X 26 cm X 26 cm dimension, each containing 5 kg of freshly procured non-sterilized field soil. The bags of each treated and untreated set-up were maintained in four replicates, in open air condition. In the amendment, vermicompost, procured from Nimpith Krishi Vigyan Kendra (West Bengal), was applied to the pot @ 100 gram per kilogram (kg^−1^) of soil. Details of the experimental design are furnished below:

*Experimental set-up 1.* SU: untreated field soil.

*Experimental set-up 2.* SU: freshly collected field soil + vermicompost.
*Experimental set-up 3.* SBC: freshly collected field soil + multi-strain bacterial consortium.
*Experimental set-up 4.* SVBC: freshly collected field soil + vermicompost + consortium.

The topsoil was covered with coco peat (1.5 cm layer) and six seeds were sown randomly in each pot. Twenty ml of the first dose of inoculant formulation was applied to the bags of the respective treated set-up near the rhizospheric region of the plants, 15 days after the seedling emergence stage, followed by two successive doses at 35- and 55-day stages, respectively. An equal amount of water was applied to each pot on every 2 days. De-weeding was practiced once in a week.

#### 2.3.2 *In vivo* plant growth promotion study

The efficacy of the amendments was tested based on their impact on selected growth characteristics of the potted plants and their yield related performance. Data were recorded every 4, 8, and, 12 weeks after the seedling emergence stage (WAE) for analyzing vegetative parameters of plants like the total number of leaves, leaf area, plant height, and the number of root nodules plant^−1^. The first onset of flowering (days) and the total number of pods plant^−1^ were recorded. After harvesting, the number of seeds pod^−1^, and the dry weight of 100 seeds were kept in record.

Chlorophyll (chl) content of leaves such as chl-a, chl-b and total chl (a + b) were measured spectrophotometrically at 4, 8, and 12 WAE, respectively. To extract chlorophyll pigment, 80% acetone was used. Freshly collected mature leaves (2nd and 3rd leaves) from the pot-grown soybean plants were used (Liang et al., 2017). The chl-a, chl-b and total chl content were estimated according to the equation of Arnon (1949):


$$\mathrm{Chlorophyll}\;a\;\left(\mathrm{\mu g}/\mathrm{mL}\right)=12.7\;\left(A_{663}\right)-2.69\;\left(A_{645}\right)\text{.}$$

$$\mathrm{Chlorophyll}\;b\;\left(\mathrm{\mu g}/\mathrm{mL}\right)=22.9\;\left(A_{645}\right)-4.68\;\left(A_{663}\right)\text{.}$$

$$\mathrm{Total}\;\mathrm{chlorophyll}\;\left(\mathrm{\mu g}/\mathrm{mL}\right)=20.2\;\left(A_{645}\right)+8.02\;\left(A_{663}\right)\text{.}$$

#### 2.3.3 Statistical analyzes

Python software version 3.11+ and its modules along with scientific computation libraries were used for plotting, analyzing, and visualizing the data obtained during the pot trial experiment. We performed two-way ANOVA on the vegetative and one-way ANOVA for reproductive parameters and subsequently conducted Tukey’s *Post Hoc* test to investigate the significant differences in the means of the observed characteristics. Both the ANOVA and Tukey’s test were conducted at a standard significance level of 5%. Normality test was performed to determine, if the concerned variables follow normal distribution pattern or not. Based on it, paired sample t-test was conducted to detect, whether the effect of combined treatment of vermicompost and bacterial consortia on different plant parameters were significant or not. To study the effect of the combined treatment of vermicompost and bacterial consortia on vegetative growth and yield related characteristics of soybean plants, the observed plant parameters in SVBC condition were compared with those of SU condition by applying paired t-test. Finally Logistic Regression mode was followed to find out if the improvement in vegetative parameters of plants such as, total no. of leaves plant^−1^ (X1), leaf area (X2), total chl content of leaves (X3) and total no. of root nodules, is reflected on the yield characteristics, which is captured best by total no of pods plant^−1^. Total no. of pods plant^−1^ (Yi) is the dependent binary variable. The empirical specification is:


Yi=α+β1X1+β2X2+β3X3+β4X4+errorterm.

Where:

Yi = 0, at vegetative state,

Yi = 1, at harvesting state.

### 2.4 DNA extraction and metagenomic sequencing of treated and non-treated field soil

The MOBIO PowerSoilTM DNA Isolation kit (Qiagen, United States) was utilized for the isolation and extraction of bacterial DNA from sieved soil samples for the execution of downstream metagenomic analyzes (Bag et al., 2016; Ghosh et al., 2022). The DNA hence obtained was sequenced on Illumina MiSeq using reagent kit V3 according to the manufacturer’s protocol for generating 2 × 300 bp paired-end reads and quality assessment was carried out using Nanodrop followed by semi-quantitative estimation of DNA via agarose gel electrophoresis. QUBIT assay was performed to obtain the precise concentration of the extracted DNA. Gene library preparation was carried out by amplifying the standardized V3-V4 region of 16SrRNA as per Illuimina gene library construction protocol.

### 2.5 Bioinformatics analysis/analyses

The sequenced raw reads were processed through the FASTQC pipeline for quality checking followed by which the screened sequences surpassing the quality threshold were finally assembled via homopolymer elimination, minimization of artefactual noise and probable contamination, using SILVAngs (1.3) pipeline (Lepinay et al., 2018). In accordance with the pipeline followed by Ganguli et al. (2017) and Mukhopadhyay et al. (2021). Operational Taxonomic Units (OTUs) were clustered using QIIME2, and microbial abundances were analyzed using KRONA charts (Estaki et al., 2020). The user-end reads yielded from Illumina sequencing were used as query sequences and subjected to the LAST algorithm for matching against the RDP_16S_18S database, for analyzing bacterial matches, at different taxonomic levels, using an alignment score cut-off of 0.8 subsequent to the elimination of reads having very high e-values. The data obtained herein was used for downstream analyzes. Starting from the widest taxonomic level, it assigned a taxonomic label to each read. The taxon that received the most hits was used for this purpose. The analysis continued until a confidence level was reached or numerous taxa were supported by the same quantity of high-quality hits. All unmatched or unclassified reads were removed from the data for downstream analyzes.

Using Krona Tools, the representative taxon was displayed in interactive graphs (Ondov et al., 2011). Data on bacterial and archaeal members were identified by using PATRIC (Wattam et al., 2017). Identification of common elements among the experimental soil under differential treatment conditions was done using Venny 2.1.0 to generate Venn Diagrams (Oliveros, 2015). An in-house algorithm has been used to integrate microbial co-inhabitance patterns and several updated datasets of different curated microbial function maps. The common genera among datasets of the field soil (S) were compared with that of soybean plant-grown untreated (SU), vermicompost-treated (SV), consortia-treated (SBC), and jointly vermicompost-consortia-treated (SVBC) soil. The results thus obtained were used to generate the Venn diagram. The enriched metabolic pathways were determined according to the Kyoto Encyclopedia of Genes and Genomes (Kanehisa and Goto, 2000). The most crucial functions were examined, and literature was mined to corroborate the data obtained as well as to correlate it with subsequent findings, including the most significant genera (as well as their inherent hierarchy) in the dataset were visualized as heatmaps, showing variations across the samples under study. The predominant functions in metabolic participation were also visualized and separated using enrichment networks. Finally, abundant genera across the samples were represented through a clustering algorithm using R code (Kolde, 2019).

## 3 Results and analysis

### 3.1 Soil nutrient status

Soil parameters like organic carbon, available potassium, nitrogen, and phosphate content were estimated, and the results are presented in Table 1. The soil was observed to have a very low level of organic carbon (0.34%) and available nitrogen (48.7 mg/kg) content according to the Indian standard (Khurana and Kumar, 2022). The estimated levels of available phosphorus and potassium were recorded as 27.25 mg/kg and 136.60 mg/kg, respectively.

**Table 1:** Nutrient status of soil sample.

Soil parameter	Result
Organic carbon content (%)	0.34 ± 0.416
Available nitrogen (mg/kg)	48.7 ± 0.135
Available phosphorus (mg/kg)	27.25 ± 0.057
Available potassium (mg/kg)	136.60 ± 0.226

All values expressed as Mean ± Standard deviation.

All values expressed as Mean ± Standard deviation.

### 3.2 Evaluation of *in vivo* growth-promotion efficacy of the PGPB consortium

**Figure 1:**
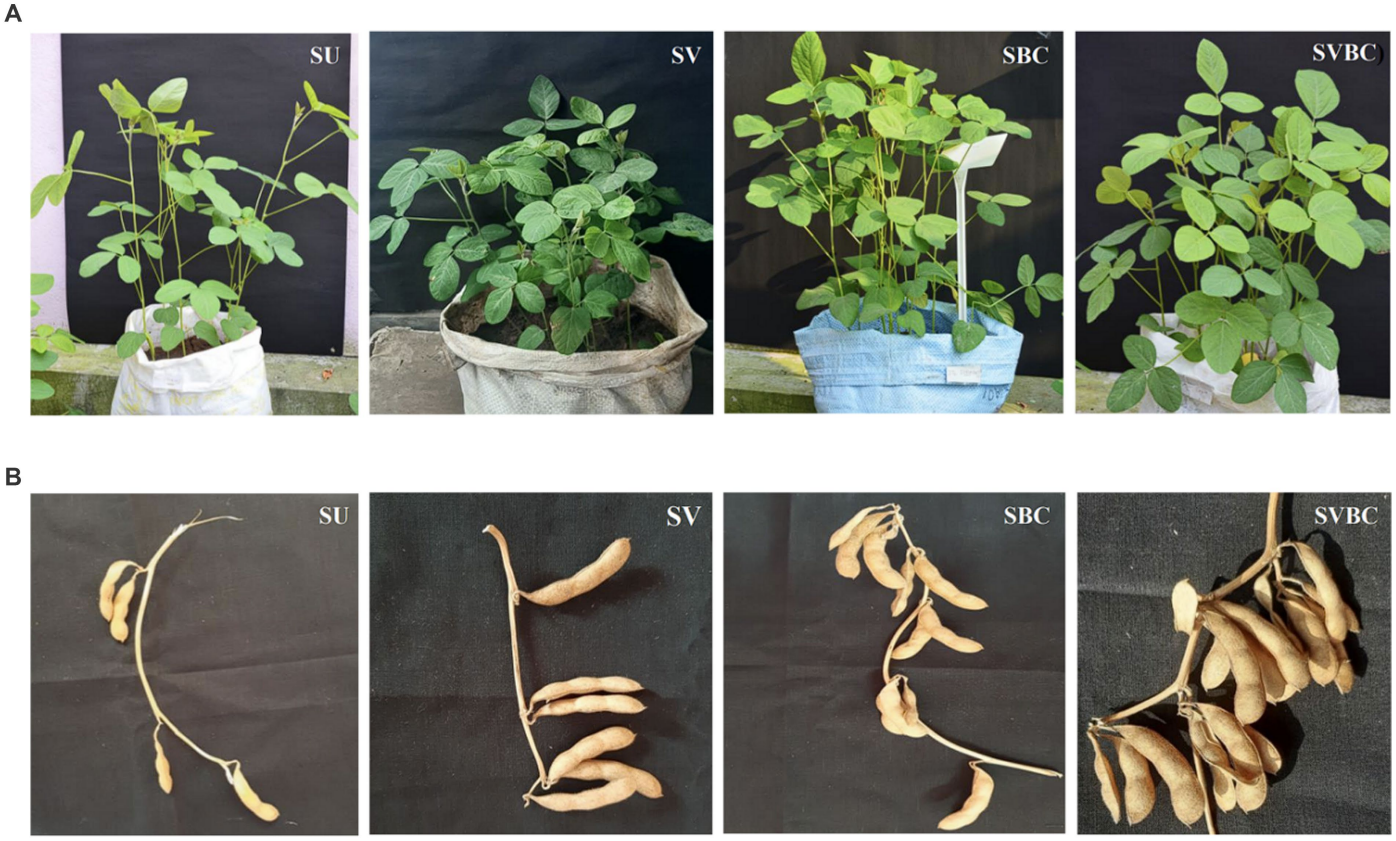
Effect of bacterial inoculum on the growth of *Glycine max* plants in different experimental set-ups. **(A)** Vegetative growth pattern of plants at 8 weeks after seedling emergence stage in treated and untreated set-ups; **(B)** A portion of twigs of untreated and treated plants show fruiting behavior.

The influence of the soil amendment on the vegetative growth, flowering, and yield-related behavior of soybean plants in different experimental conditions (SU, SV, SBC, and SVBC) was analyzed. Varying effects were observed at 4, 8, and 12 WAE stages of the plant growth with respect to vegetative growth characteristics like leaf density, leaf area, plant height, and nodule numbers. The non-inoculated plants grown in the SU set-up were inferior with respect to most the parameters studied, whereas the highest improvement was recorded in bacterial consortium-inoculated plants grown in vermicompost--treated soil (SVBC) followed by SBC condition (Figure 1).

**Figure 2:**
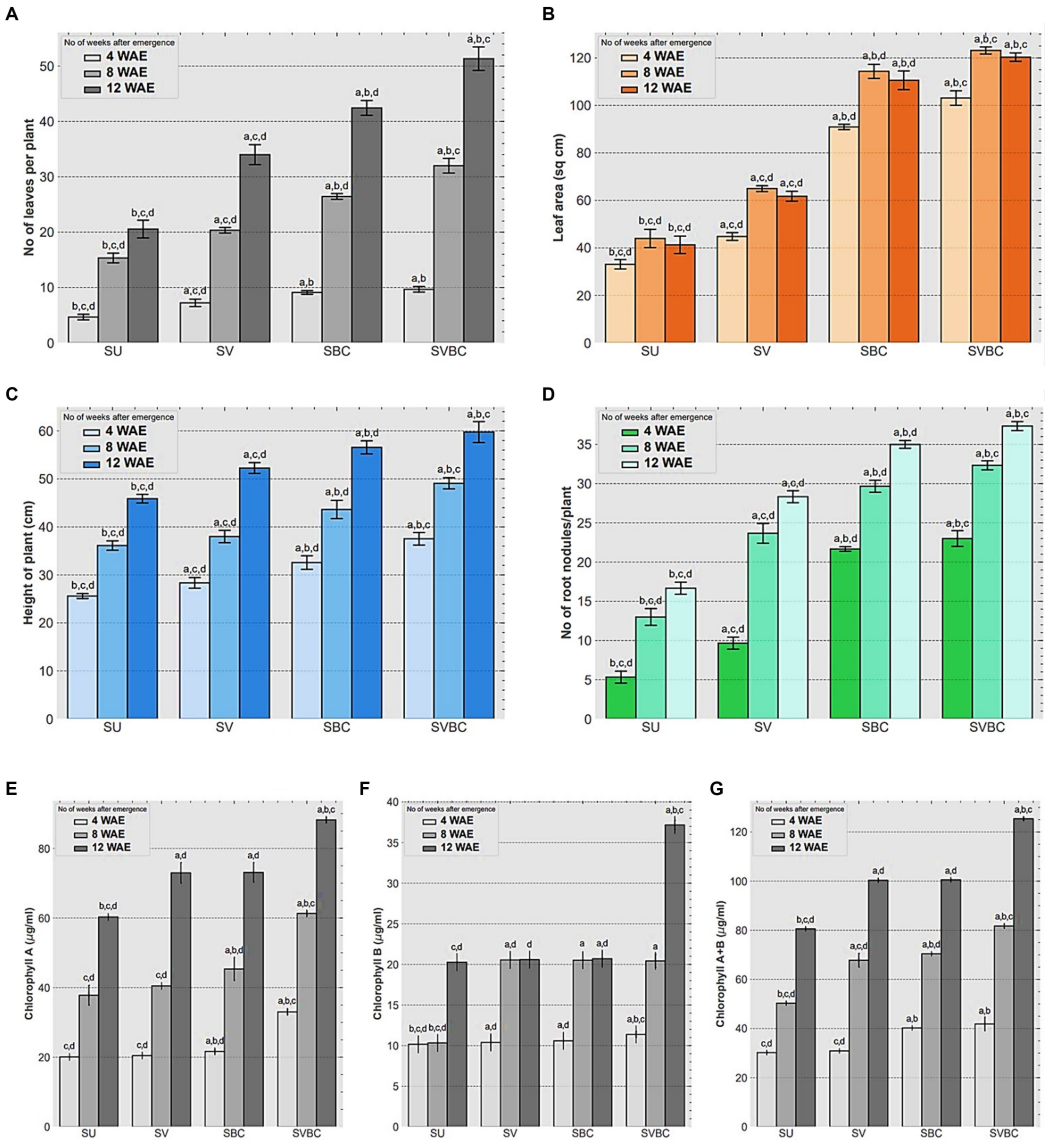
Effect of different treatments on vegetative parameters and chlorophyll content of leaves of *Glycine max* Merill. plants at 4, 8, and 12-week stages after seedling emergence (WAE). **(A)** Number of leaves per plant; **(B)** Leaf area; **(C)** Height of plants, **(D)** Number of root nodules per plant; **(E)** Chlorophyll-a content of leaves; **(F)** Chlorophyll-b content of leaves; **(G)** Total Chlorophyll content of leaves. SU, untreated soil; SV, soil amended with vermicompost; SBC, treated with the bacterial consortium; SVBC, soil amended with vermicompost and bacterial consortium. Columns represent the mean values of the data for each characteristic and the error bars represent the standard deviation. Different letters on columns imply the significant difference between the means of the data (*p* < 0.05) as evaluated by Tukey’s HSD test after a one-way ANOVA test.

The findings of vegetative growth-related traits of the plants at different experimental conditions are furnished in Figure 2. The total number (mean) of leaves plant^−1^ observed, at 12 WAE stages in SU, SV, SBC, and SBVC set-ups were 22, 34, 43, and 51, respectively (Figure 2A). Leaf area (mean) increased remarkably in all the treated experimental set-ups at stages over to that of the un-inoculated control condition, showing most striking improvement in at the 12 WAE stage of the plants, ranging from 40.2 sq. cm in SU to 61.7 sq. cm, 99.8 sq. cm, and 102.5 sq. cm in SV, SBC, and SVBC conditions, respectively (Figure 2B). Enhancement in plant height was evidenced by 14.13, 21.73, and 32.6% increase in the SV, SBC, and SVBC set-ups at 12 WAE, and a similar trend was recorded at 4 and 8 WAE stages (Figure 2C). The root nodule number was significantly high in SBC and SBVC set-ups in comparison to the other set-ups (Figures 2E,F). Total chlorophyll content leaves showed a significant increase by 24.6%. 25.6%, and 55.4%, respectively under SV, SBC, and, SVBC conditions, at 12 WAE stage over to that of the untreated one (Figure 2G). The overall trend indicates an enhancement in plant performance following soil amendment with only vermicompost, only bacterial inoculant and a combined treatment with vermicompost-consortium. However, the most promising result was observed in SBVC condition.

**Figure 3:**
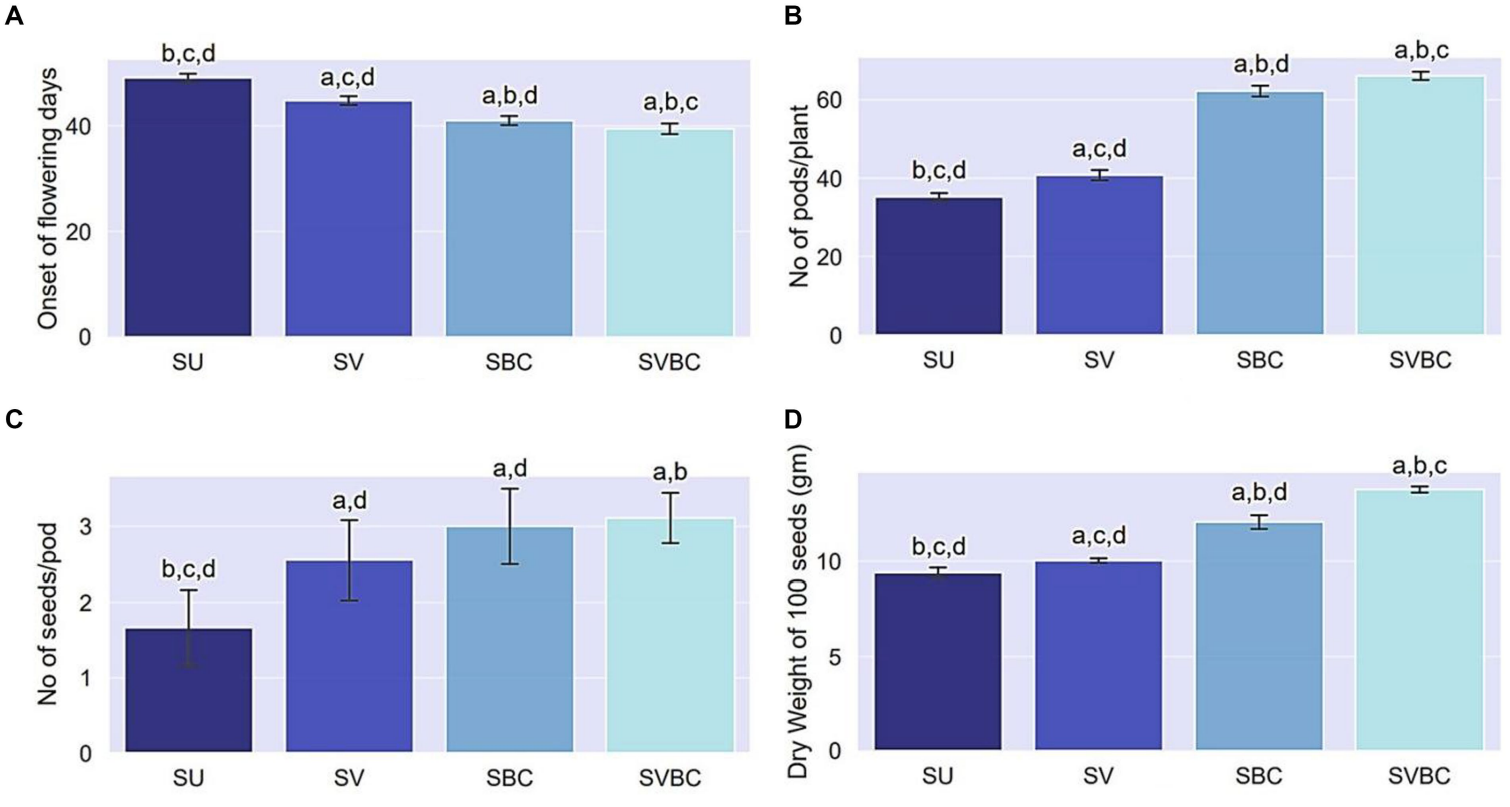
Effect of different treatments on reproductive and yield attributes of *Glycine max* Merill. Plants. **(A)** First onset of flowering (days); **(B)** No. of pods plant^−1^; **(C)** No. of seeds pod^−1^, **(D)** Weight of 100 seeds. Columns represent the mean values of the data for each characteristic and the error bars represent the standard deviation. Different letters on columns imply the significant difference between the means of the data (*p* < 0.05) as evaluated by Tukey’s HSD test after a one-way ANOVA test. SU, untreated soil; SV, soil amended with vermicompost; SBC, treated with the bacterial consortium; SVBC, soil amended with vermicompost and bacterial consortium.

The pot trial experiment showed a significant improvement in reproductive and yield-related traits of the soybean plants following amendment practices (Figure 3). Soil augmentation with the joint-treatment of vermicompost and the novel consortium, exerted a positive effect on the initial blossoming stage as evidenced by 8.1, 16, and 20.4% decrease in the first onset of flowering days in SV, SBC, and SVBC in comparison to that of SU set-up indicating early flowering in consortium treated conditions (Figure 3A). Combined application of vermicompost and bacterial consortium inoculant tremendously influenced the number of pods node^−1^ (Figure 3B) and consequently, on the total number of pods plant^−1^, which was highest in SVBC condition (88.1% increase) compared to the SU set-up (Figure 3B). An increase in the number of seeds pod^−1^ was also evident in inoculant-treated soil, both with and without vermicompost supplementation conditions (Figure 3C). Application consortium incredibly increased the dry weight of seeds by 24.46 and 45.7% in SBC and SBVC set-ups, respectively whereas in vermicompost-enriched soil, it improved by only 7.4% (Figure 3D).

During statistical analysis, Tests of Normality showed that, all the concerned variables related to plant growth and performance followed normal distribution as confirmed by Shapiro–Wilk test. The test statistics were not significant at 5% level (Table 1; Supplementary Table S1).

Hence, we proceeded for t-test which is appropriate for this study. It was observed that t-statistic was highly significant at 5% level which confirmed that there has been significant improvement in the total no. of leaves plant^−1^, total no. of pods plant^−1^, total no. of chlorophyll content of leaves, total no. of root nodules plant^−1^, and dry weight of 100 seeds in SVBC condition over to that of SU condition (Supplementary Table S2). Finally, Logistic regression model indicates that, if there is per unit rise in the no. of leaves plant^−1^ (X_1_), leaf area (X_2_), total chl content of leaves (X_3_) and total no. of root nodules, then there is likelihood that the no. of pod plant^−1^ will increase by 17.91, 17.86, 17.98, and 17.17.99 units, respectively (Table 2).

**Table 2:** Logistic regression.

Step 0	Variables		Score	df	Sig.
		No_leaves	17.913	1	0.000
		Leaf_area	17.869	1	0.000
		Chl_content	17.984	1	0.000
		Root_nodules	17.631	1	0.000
	Overall statistics		17.988	4	0.001

### 3.3 Insights into bacterial abundance in treated and untreated soil

The comprehensive analysis of our investigation was centered around field soil samples obtained from five unique environmental conditions. Each specific condition or environment was meticulously segregated and processed as an individual dataset, with the detailed specifications of each outlined in Table 3. For researchers and practitioners who are keen on delving deeper into the methodological specifics and ensuring data integrity, the Quality Control (QC) Parameters associated with the Paired-End Miseq Illumina Sequences for these datasets can be perused in Supplementary Table S3.

**Table 3:** Sample nomenclature codes and NCBI SRA accession number.

Data set	Sample code	Experimental condition	Analysis code	NCBI SRA Project: PRJNA689214
				SRA accession number
1	S	Field Soil	SAM3	SRX 9768638
2	SU	Field Soil+Soybean Plant	SAM2	SRX 9815238
3	SV	Field Soil + Vermicompost + Soybean Plant	DHB3	SRX 19133782
4	SBC	Field Soil + Bacterial Consortium+Soybean Plant	DHB1	SRX 19133762
5	SVBC	Field Soil+Vermicompost +Bacterial+Sybean plants	DHB2	SRX 19133763

Direct link to the data submitted to NCBI SRA: https://www.ncbi.nlm.nih.gov/sra/PRJNA689214.

Upon an initial examination of the microbial diversity profiles across the datasets, certain patterns and disparities come to the fore. Most notably, the 5th dataset, designated as SVBC (an amalgamation of field soil that was treated with the combined efforts of vermicompost, bacterial consortia, and *in situ* soybean plants), presented an intriguing dichotomy. On the surface, its alpha diversity metric–a fundamental measure that encapsulates the richness and diversity within a singular ecosystem–was observed to be slightly subdued when juxtaposed with the 4th dataset. This might lead one to hastily surmise that SVBC was less diverse or less robust than its counterparts. However, a more nuanced metric, the Shannon diversity index, paints a contrasting picture. This index, which serves as a reliable barometer for the species diversity within an ecosystem, recorded a higher value for the SVBC dataset, as captured in Table 4. Such an observation insinuates that the SVBC environment, while potentially having fewer genera than others, exhibited a broader and more uniform distribution of those genera present. Such a balanced and evenly spread microbial community could be indicative of a robust and harmonized ecosystem. It implies that the SVBC condition may host a microbial environment that, while not the most varied, is characterized by stability, resilience, and an intricate balance of microbial interactions.

**Table 4:** Alpha and Shannon diversity indices.

Data set	Sample code	Alpha diversity	Shannon diversity index
1	S	228	2.418
2	SU	368	3.142
3	SV	281	3.439
4	SBC	377	3.426
5	SVBC	328	3.496

This interplay and uniformity might have implications for soil health, plant growth, and overall ecosystem stability, warranting further in-depth studies and explorations into the underlying mechanisms and benefits.

#### 3.3.1 Dataset 1: soil sample code: S

**Figure 4:**
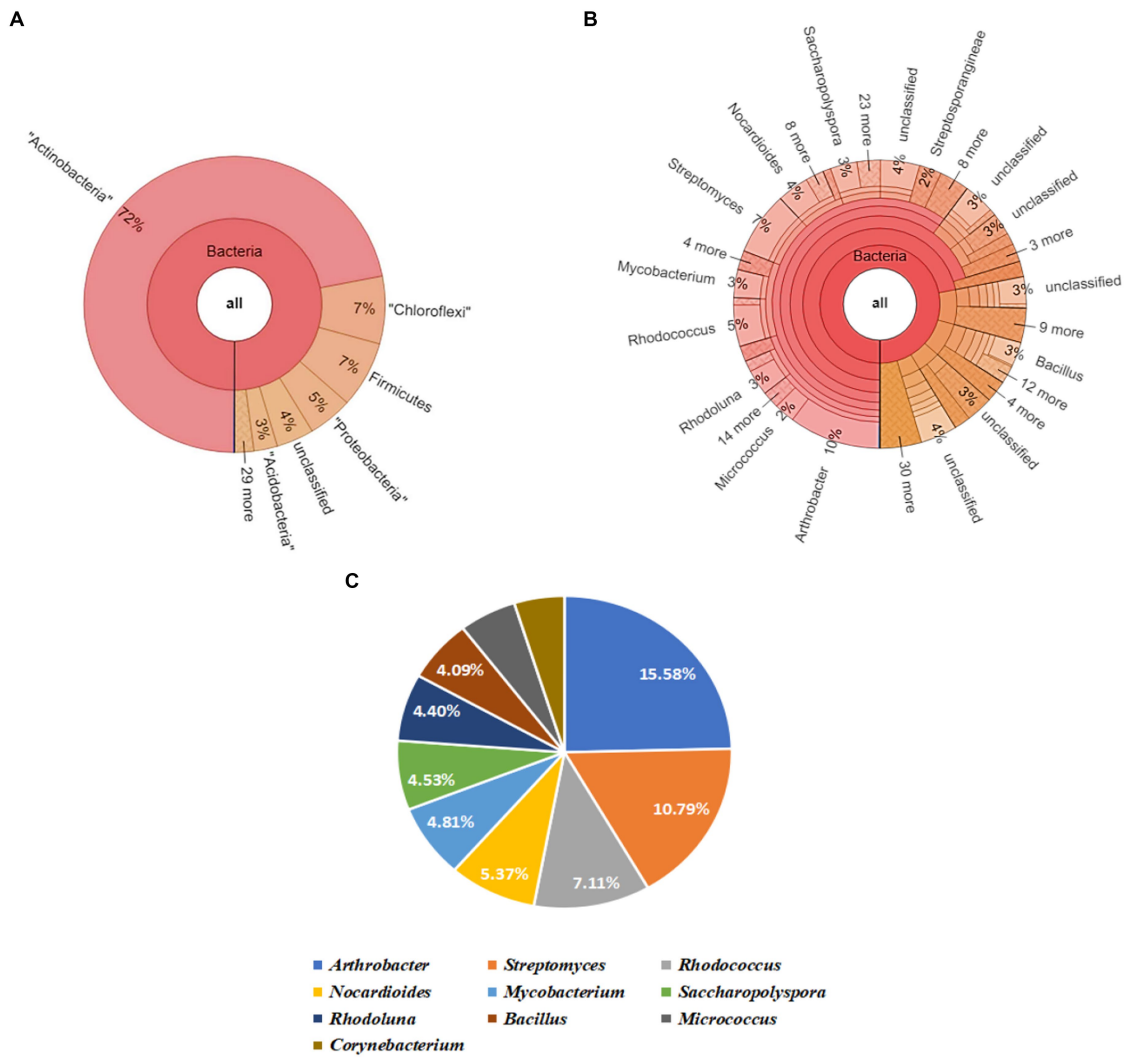
Bacterial abundance in S (untreated field soil) condition. **(A)** Krona chart representation of Phyla level abundance of prevalent bacterial assemblage; **(B)** Krona chart representation of Genera level abundance of prevalent bacterial assemblage; **(C)** Pie chart representing the top 10 scoring genera.

The Krona map yielded by running the raw sequence reads through a suitable pipeline revealed that Actinobacteria was the most abundant phylum, followed by Chloroflexi, Firmicutes, Proteobacteria, and Acidobacteria in soil sample (S). Upon further screening of the putative top 10 genera that were most abundant in the given sample, *Arthrobacter* and *Streptomyces* had relative abundances of 15.58 and 10.79% (Figure 4).

#### 3.3.2 Dataset 2: soil sample code: SU

**Figure 5:**
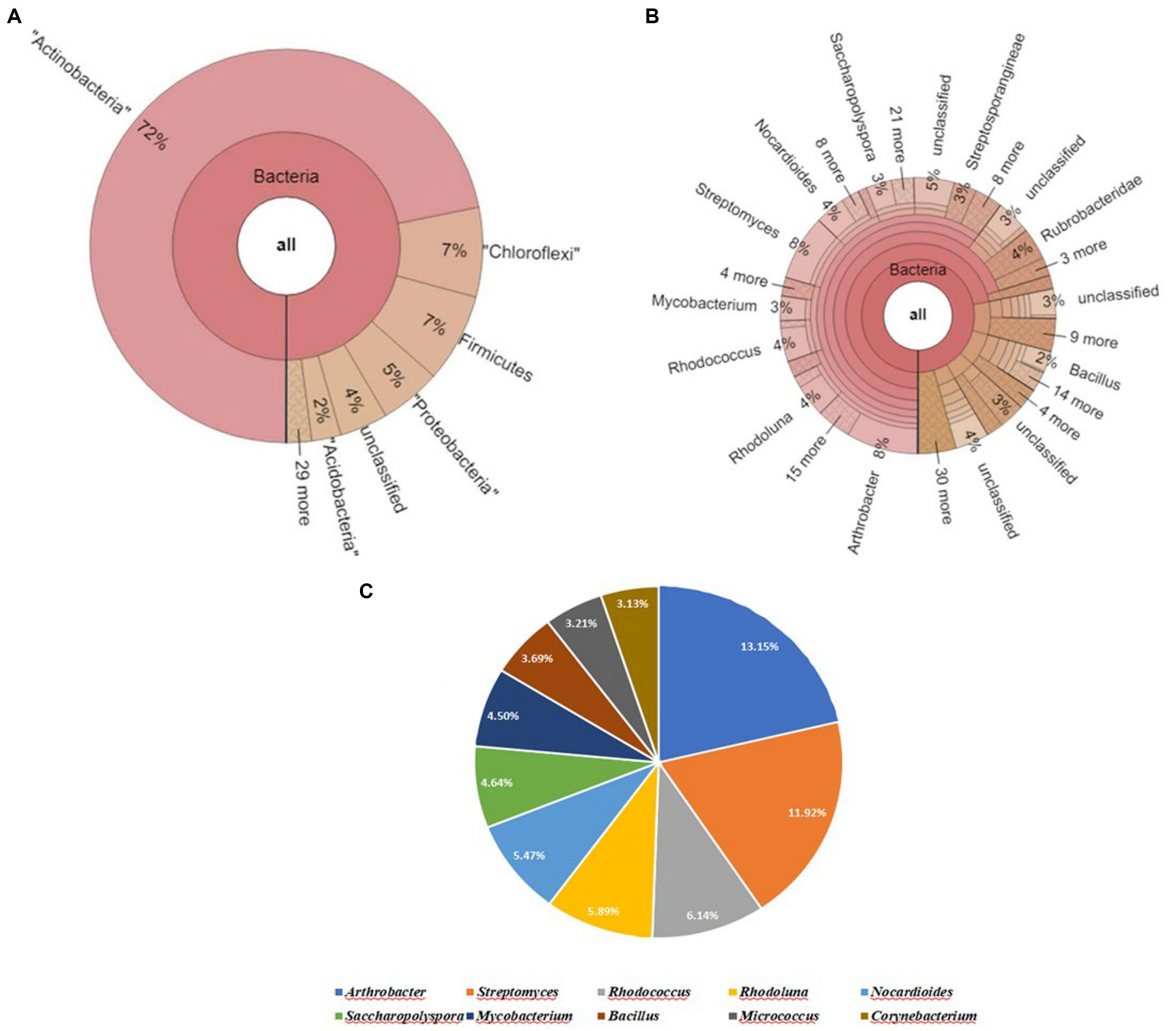
Bacterial abundance in SU (Untreated field soil with soybean plants) condition. **(A)** Krona chart representation of Phyla level abundance of prevalent bacterial assemblage, **(B)** Krona chart representation of Genera level abundance of prevalent bacterial assemblage, **(C)** Pie chart representing the top 10 scoring genera.

It was found that Actinobacteria was the most abundant phylum, followed by Chloroflexi, Firmicutes, Proteobacteria, and *Acidobacteria*. The untreated field soil sample in the presence of soybean plants (SU) contained the following top 4 genera: *Arthrobacter* and *Streptomyces* (8%), *Rhodococcus* (4%), *Mycobacterium* (3%) and *Bacillus* (2%). Upon further screening of the putative top 10 genera that were most abundant in the given sample were *Arthrobacter* and *Streptomyces* had relative abundances of 13.15 and 11.92% (Figure 5).

#### 3.3.3 Dataset 3: soil sample code: SV

**Figure 6:**
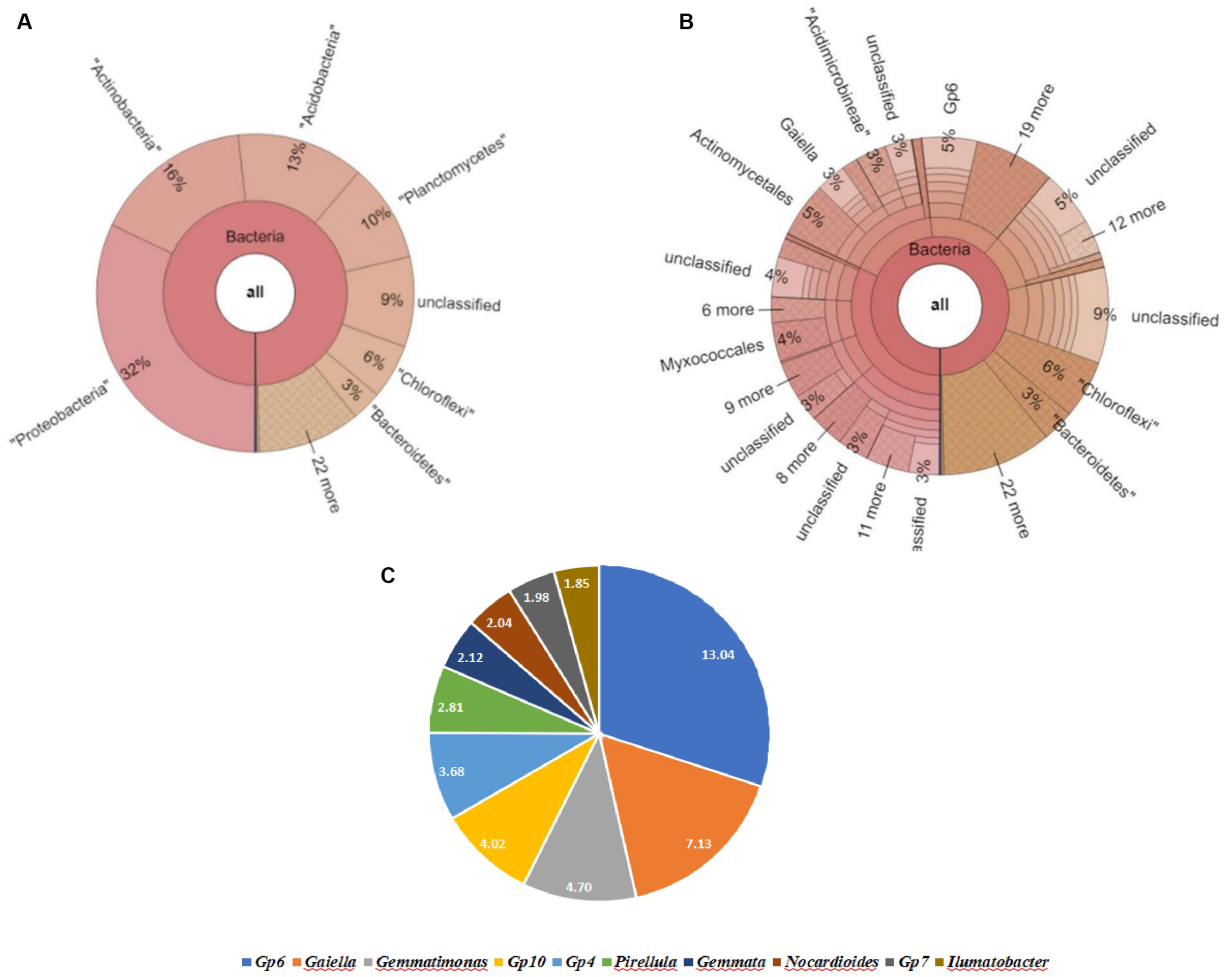
Bacterial abundance in SV (Field soil treated with vermicompost + soybeanplants) condition. **(A)** Krona chart representation of Phyla level abundance of prevalent bacterial assemblage, **(B)** Krona chart representation of Genera level abundance of prevalent. **(C)** Pie chart representing the top10scoring genera.

The Krona map revealed that Actinobacteria appeared to be the most abundant phylum, followed by Acidobacteria, Planctomycetes, Chloroflexi, Bacteroidetes, and Proteobacteria in the soil sample SU. It included the following top 4 genera: Gp6 (5%), *Gaiella* (3%), *Gemmatimonas* (2%), and Gp10 (2%) (Figure 6).

#### 3.3.4 Dataset 4: soil sample: SBC

**Figure 7:**
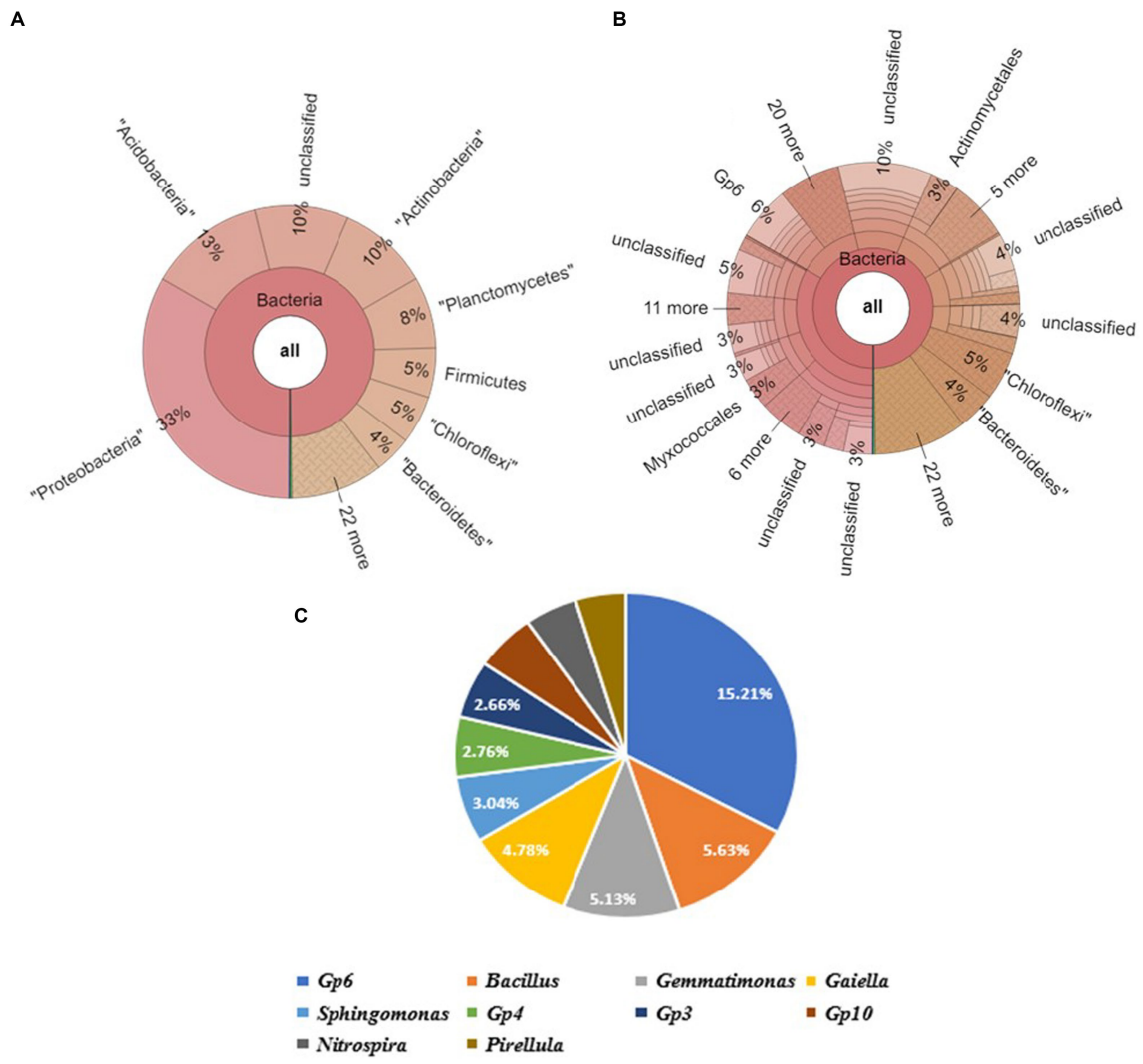
Bacterial abundance in SBC (Field soil treated bacterial consortium+soybean plant) condition. **(A)** Krona chart representation of Phyla level abundance of prevalent bacterial assemblage, **(B)** Krona chart representation of Genera level abundance of prevalent bacterial assemblage, **(C)** Pie chart representing the top 10 scoring genera.

Proteobacteria was observed to be the most abundant phylum, followed by Acidobacteria, Actinobacteria, Planctomycetes, Firmicutes, Chloroflexi, and Bacteroidetes in soil sample SV. It contained of the following top 4 genera: Gp6 (6%), *Bacillus* (3%), *Gemmatimonas* (2%), and *Gaiella* (2%). Upon further screening of the putative top 10 genera recorded to be the most abundant in the given sample, *Gp6* and *Bacillus* have relative abundances of 15.21 and 5.63% (Figure 7).

#### 3.3.5 Dataset 5: soil sample code: SVBC

**Figure 8:**
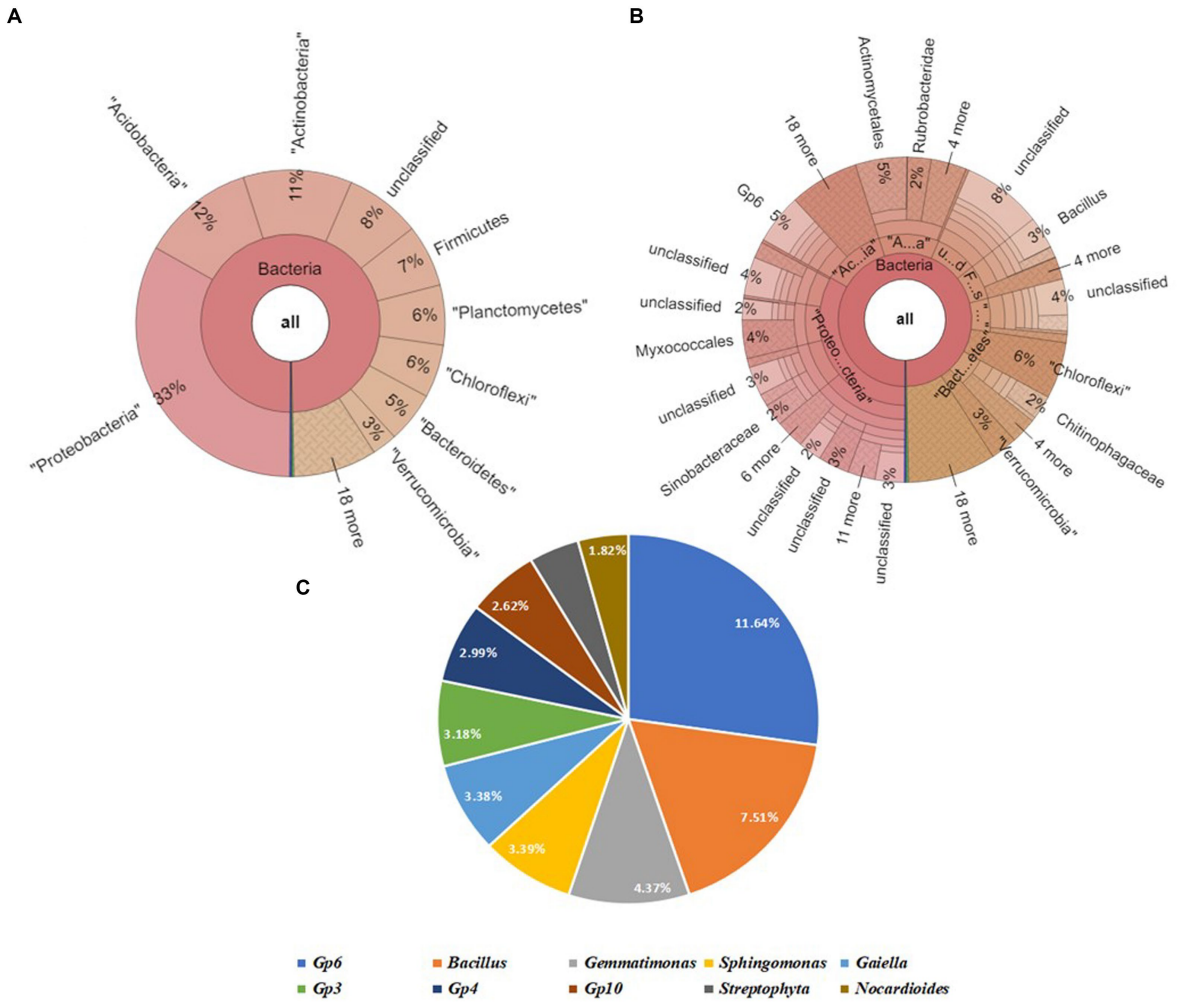
Bacterial abundance in SVBC (Field soil treated with vermicompost +bacterial consortium + soybean plants) condition. **(A)** Krona chart representation of Phyla level abundance of prevalent bacterial assemblage, **(B)** Krona chart representation of Genera level abundance of prevalent bacterial assemblage, **(C)** Pie chart representing the top 10 scoring genera.

The Krona map revealed that Proteobacteria was the most abundant phylum, followed by Acidobacteria, Actinobacteria, Firmicutes, Planctomycetes Chloroflexi, Bacteroidetes, and Verrucomicrobia. The soil sample SBC contained the following top 4 genera: Gp6 (6%), *Bacillus* (3%), *Gemmatimonas* (2%), and *Sphingomonas* (2%). Upon further screening of the putative top 10 genera that were most abundant in the given sample, Gp6 and *Bacillus* have relative abundances of 11.64 and 7.51% (Figure 8).

### 3.4 Comparative profiling of treated and untreated soil samples

**Figure 9:**
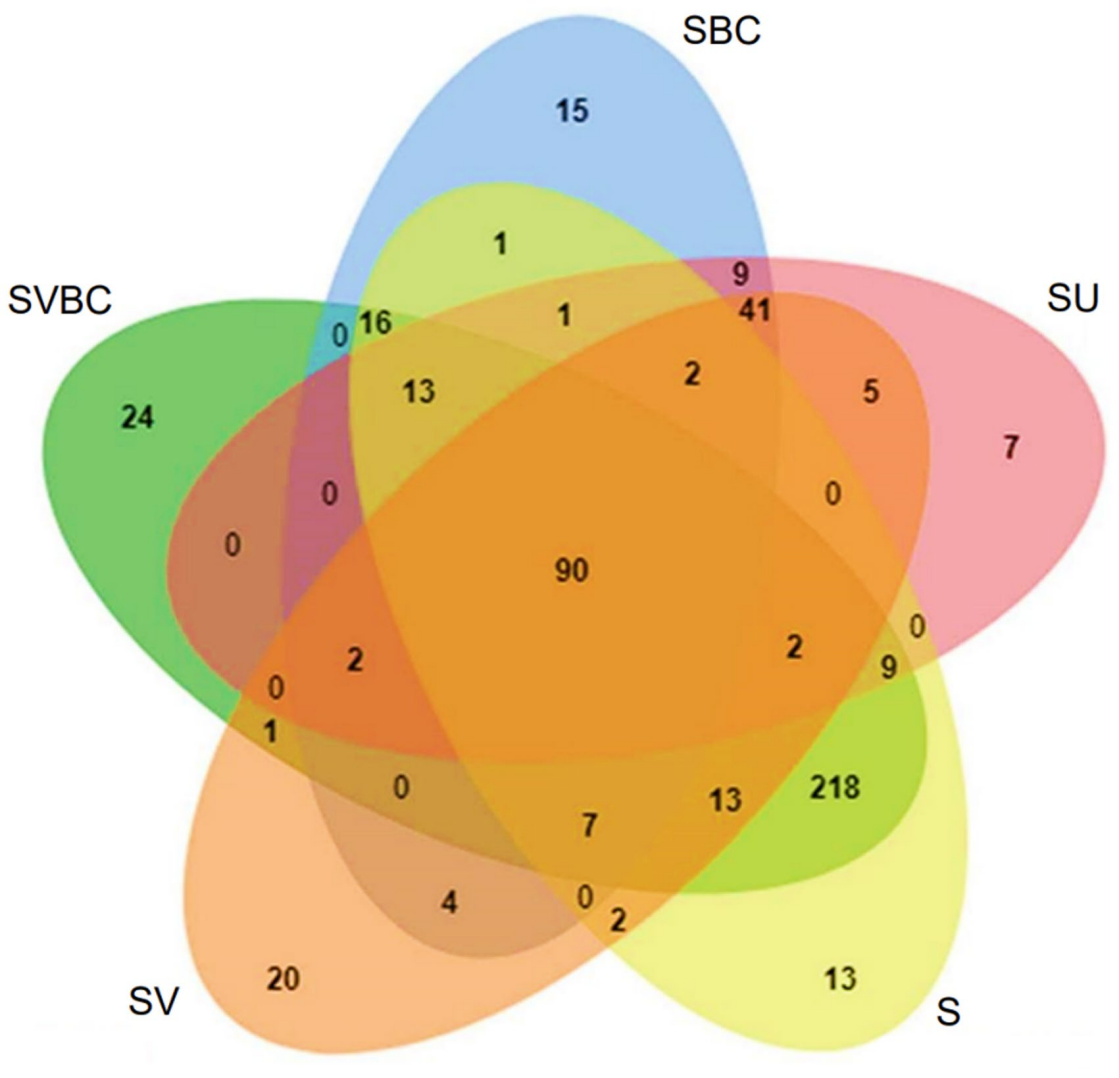
Comparative Venn diagram depicting the common and unique bacterial members among the five samples under study.

Some of the bacterial genera common across all the datasets spanning varying ranges of treatments were *Rhizobium*, *Bradyrhizobium*, *Mesorhizobium*, *Microbacterium*, *Paenibacillus*, *Bacillus*, and *Pseudomonas*. The bacterial genera unique to the untreated field soil sample were *Neptuniibacter*, *Lysinimonas*, *Alcanivorax*, *Campylobacter*, *Neisseria*, *Methylococcus*, and *Oceanobacillus*. The set of bacteria that were found to be unique to the soil sample SU, includes *Anaerotruncus*, *Dialister*, *Rhodoferax*, *Parvimonas*, *Negativicoccus*, *Hoeflea*, and *Ruegeria*. The soil sample in SV condition, revealed a unique set of bacterial genera which include *Rhodoplanes*, *Neochlamydia*, *Byssovorax*, *Thermogutta*, *Verrucomicrobium*, *Luedemannella*, and *Tahibacter*. When exposed to the treatment with the defined bacterial consortium, the field soil sample in SBC condition exhibited a unique bacterial profile consisting of *Thauera*, *Ignavibacterium*, *Thermoactinomyces*, *Solitalea*, *Syntrophobacter*, *Fluviicola* and *Solimonas*. Under the concerted application of vermicompost and the bacterial consortium (SVBC), a unique bacterial profile was isolated from the experimental soil which included *Rhodanobacter*, GpV, *Clostridium*, *Okibacterium*, *Dokdonella*, *Phycicoccus*, and *Pedobacter*. The comparative Venn diagram of the common and unique bacterial members among the five samples under study, depicts that the enriched soil shows a higher number of unique members indicative of an improvement in overall soil health thus, indicating promotion of more and more associative microbial assemblage (Figure 9).

**Figure 10:**
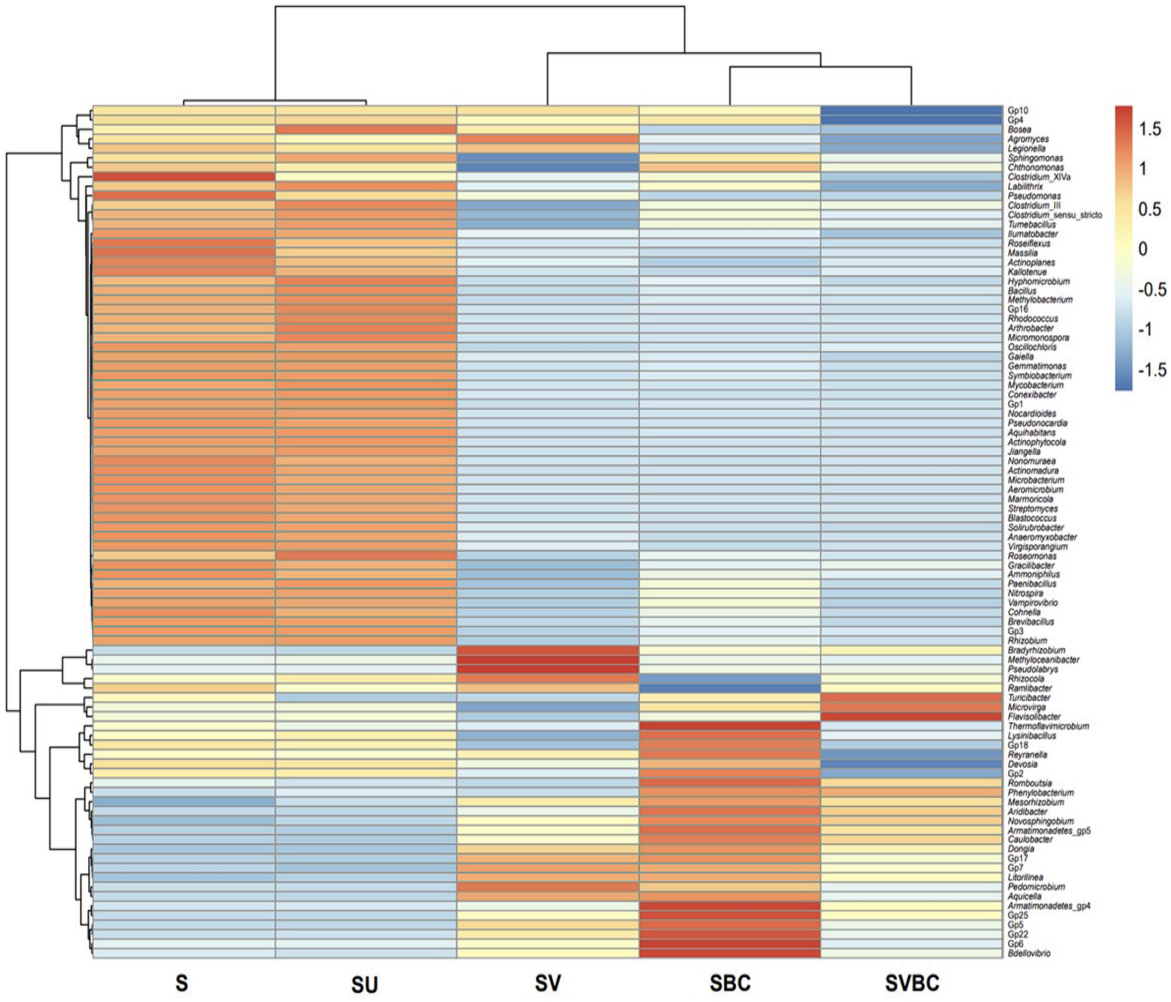
Heatmap depicting the abundance of important growth-promoting bacterial members in the five samples under study.

The functional genera in the field soil sample treated jointly with vermicompost and selected bacterial consortium in the presence of soybean plant (SVBC) showed the highest level of enrichment. However, important PGPB genera show an intermediate level of abundance in the case of the soil samples treated with bacterial consortia (SBC) and vermicompost (SV), independently. The untreated field soil (S) and soil sample SU exhibited the lowest level of abundance in the functional genera (Figure 10).

### 3.5 Functional profiling of the soil sample under varying experimental conditions

**Figure 11:**
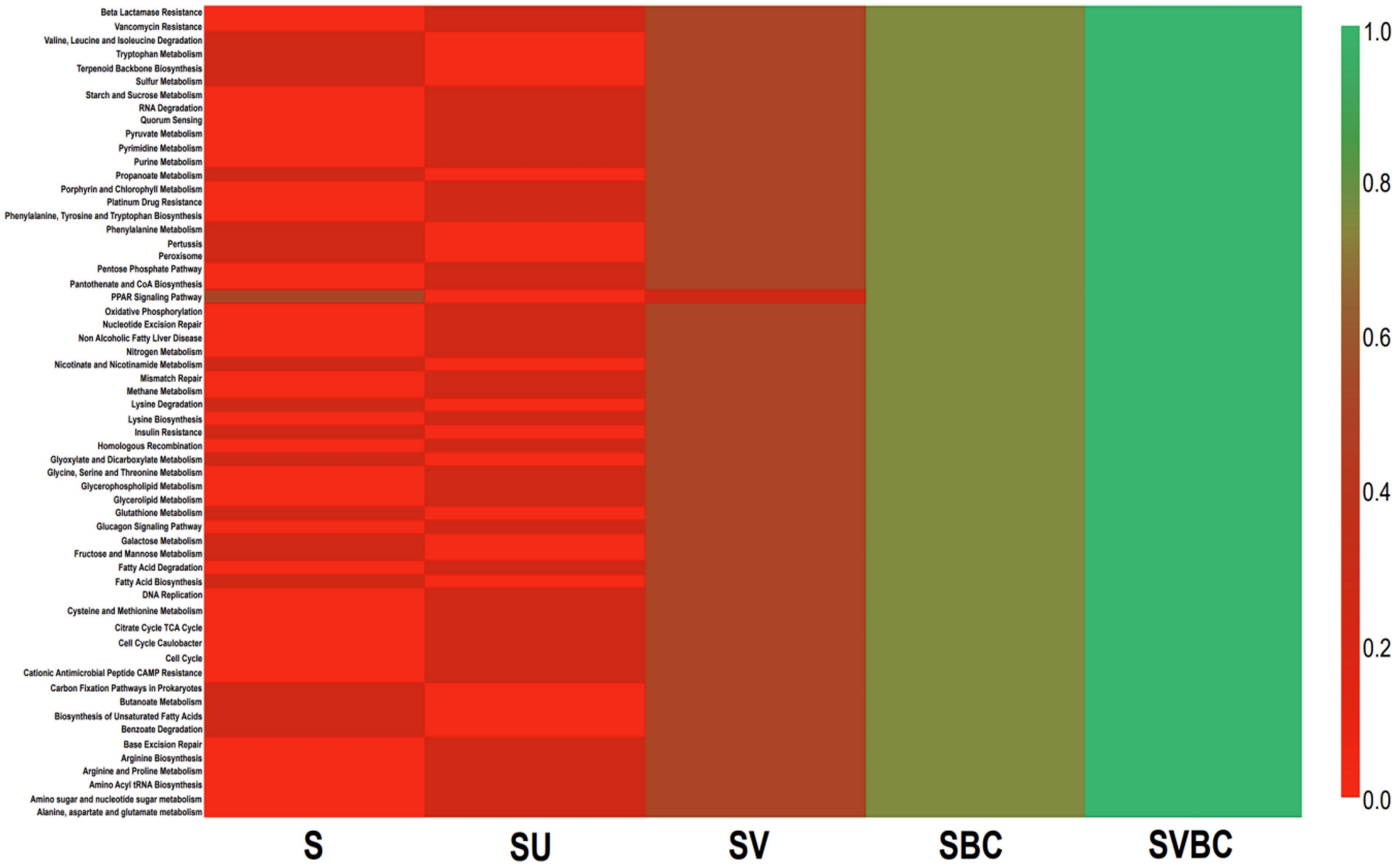
Heat map of functional profiling showing variations in the predicted active pathways of the common genera in the field soil obtained from controlled and experimental conditions.

Further downstream analysis of the target genera like *Rhizobium*, *Bradyrhizobium*, *Mesorhizobium*, *Microbacterium*, *Paenibacillus*, *Bacillus*, and *Pseudomonas* reveals a stringent relationship in terms of the abundance of growth-promoting bacterial members which can be interpreted to be indicative markers across the soil samples under study. A large number of biological pathways, both homeotic and response, were predicted. The common metabolic pathways found to be of highest prevalence across the soil samples under differential experimental parameters under study were tryptophan metabolism and terpenoid backbone synthesis for soil S; starch and sucrose metabolism and quorum sensing in SU; valine, leucine, and isoleucine degradation for SV, and all the enriched metabolic cascades were found to be uniformly active in SBC or SBVC condition. Among the five datasets, SBVC exhibited a significantly higher magnitude of activation of the most prevalent functional pathways as compared to the remaining datasets in the presented heat map (Figure 11).

**Figure 12:**
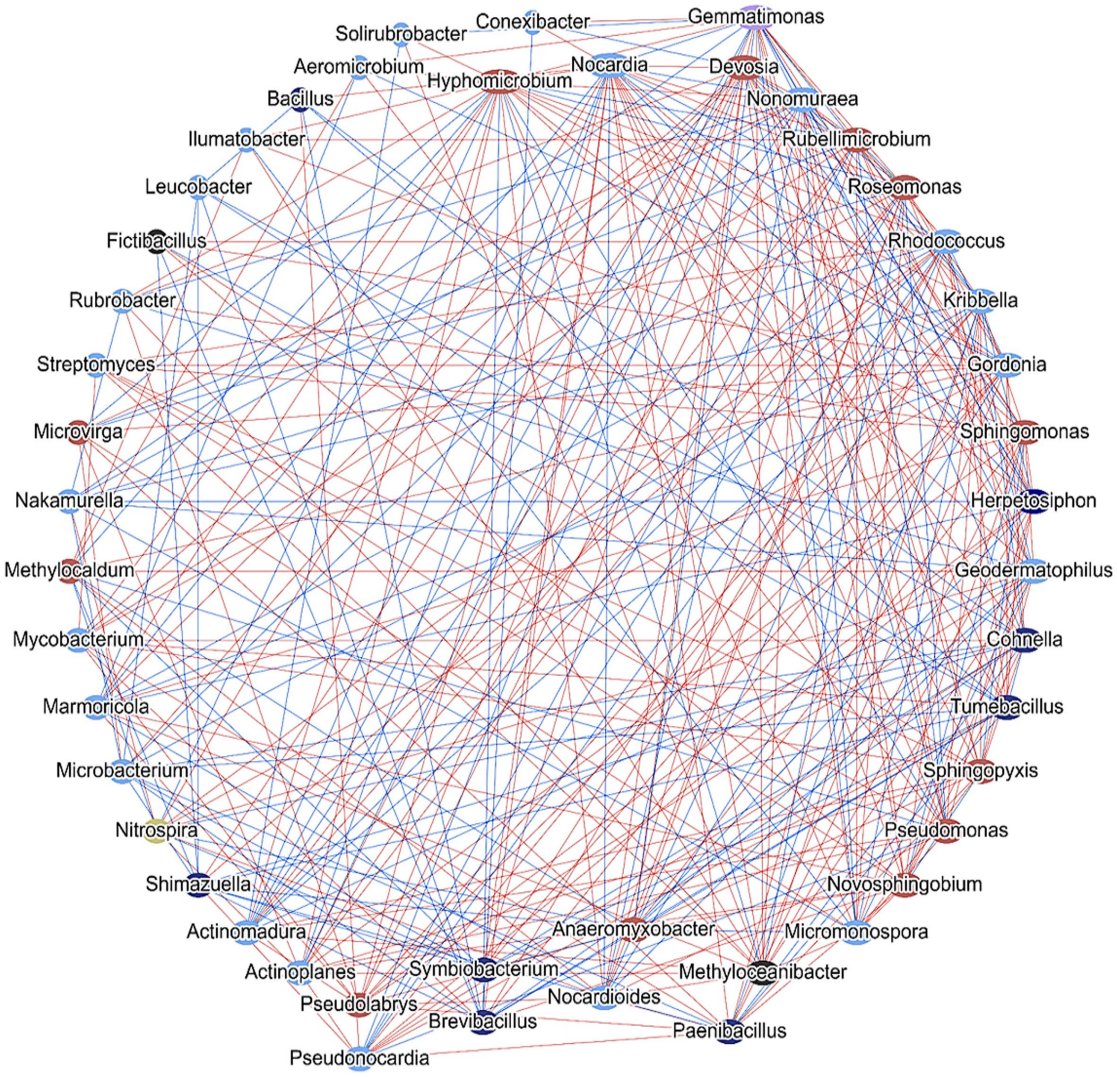
Network analysis showing cross-talk between different genera in the field soil consortia obtained from controlled and experimental conditions. Blue lines and red lines indicate bidirectional and unidirectional interactions.

The intricate web of soil microbial interactions is pivotal in defining the ecological and functional attributes of an ecosystem. Within this matrix, the soil microbial network analysis of common genera provides a comprehensive insight into the dynamic relationships these microbes maintain. This network, which presents both uni-directional (where one microbe influences another without reciprocal action) and bi-directional (mutually beneficial or antagonistic interactions) connections, captures the interdependence and synergy of microbial populations. Delving deeper into this network, functional correlation provides a more nuanced understanding. In this layout, the nodes symbolize distinct microbial entities, while the connecting edges signify shared functional contributions. This means that two connected microbes are likely collaborating or competing in some capacity, potentially impacting specific metabolic pathways or ecological functions. For instance, two microbes might co-contribute to nitrogen fixation, or one might produce a substrate that the other utilizes. The referenced Figure 12 would likely present a visual representation of this network, highlighting the complexities and nuances of these interactions. Such networks not only help in understanding the current microbial dynamics but also in predicting how changes in one microbial population might impact others. This information is essential for soil health, plant growth, and broader ecosystem stability, especially in the context of environmental changes and sustainable agricultural practices.

## 4 Discussion

Microorganisms are, without a doubt, essential pillars of our ecosystem, demonstrating resilience by flourishing in a range of environmental conditions. Their versatility and adaptability have been highlighted by key international bodies such as the United Nations General Assembly (UNGA) Science Summit. Soil is the abode of myriad of microbial communities that encompass a bewildering array of physiological, metabolic, and genomic diversity essential for sustenance of soil fertility. Over-exploitation of arable lands with persistent application of agro-chemicals, inadequate return of organic matter to cultivated land, monoculture, and soil erosion, have been negatively impacting soil structural and functional properties resulting in depletion of nutrients, lowering in microbiological diversity, soil fertility, and crop productivity (Huang et al., 2019). Sustainability of agricultural systems has become a major challenge across the world as well as, in India where 54.6% of the total workforce depends on agricultural sector for their livelihood. Literature mining indicates that restoration of microbial diversity may help to recover such damaged agro-ecosystem replenishing various plant-beneficial services at community-level, consequently, improving plant health (Delgado-Baquerizo et al., 2016). Among the BSMs, plant growth promoting bacteria (PGPB) can directly influence plant structure and performance through mobilization of soil nutrients, secretion of plant beneficial secondary metabolites (such as phytohormones, siderophore) as well as indirectly protect plants from biotic and abiotic stresses (Gouda et al., 2018). Furthermore, they are involved in maintaining physical, chemical and biological health of soil. Beneficial soil microbes are assumed to steer the bio-based revolution in the near future as a potential alternative to complement or replace chemical fertilizers and pesticides (Manfredini et al., 2021; Malusà et al., 2021). The introduction and promotion of Nature Based Solutions (NBS) echo one of the several facets of the UN SDGs to fight some of the world's greatest challenges like poverty and hunger (FAO, 2022). In the present investigation, three potent PGPB, *Bacillus subtilis* strain MMAM, *Bacillus zhanzhouensis* strain MMAM, and *Bacillus cereus* strain MMAM, isolated from an over-used arable land, were used as a composite bio-inoculant for plant-growth promotion study in the soil from the same nutrient-depleted field. These three bacterial isolates possessed multifarious PGP activity such as mineral nutrient (NPK) harnessing ability, production of IAA, GA, ACC deaminase, siderophore, and biofilm, several lytic enzymes, HCN, and NH_3_ in *in vitro* conditions (Mukhopadhyay, 2022). The effects of the inoculum on the growth promotion of soybean plants in vermicompost-treated and untreated field soil were evaluated. Furthermore, the implications of the amendments on the resident soil bacterial community were analyzed. As per the pot trial experiment, a significant improvement with respect to vegetative and reproductive parameters of the test plant were observed in varying degrees in amended conditions. The highest improvement was recorded in SBVC followed by SBC and SV set-up. Earlier researchers showed that improvement in crop yield can be achieved through indigenous or inoculated PGPB via enhanced nutrient availability or phytohormones production (Becker et al., 2018; Bechtaoui et al., 2020). The *de novo* biosynthesis of plant growth enhancers (such as cytokinins and IAA) synergistically reinforces the phytohormone signaling cascades thereby, augmenting host tolerance to various biotic and abiotic stresses from the environment that they are constantly subjected to Naveed et al. (2015). Many soil bacteria are able to produce a plethora of hydrolytic enzymes, which are directly associated with the mineralization of organic materials thus, facilitating the nutrient mineralization and carbon cycling process (Sinsabaugh et al., 2008). Positive impacts on plant growth due to *Bacillus*-induced enhanced nutrient acquisition and hormonal modulations following treatment with *Bacillus*-based formulations have been observed in recent studies (Tsotetsi et al., 2022). Furthermore, Hu et al. (2021) reported that application of multi-strain microbial consortia inoculants (*Pseudomonas* spp.) is capable of enhancing plant growth more effectively compared to that of single-strain inoculants. Co-inoculation of *Glycine max* L. plants with *Bradyrhizobium japonicum* and *Azospirillum brasilense* inoculants showed outstanding results for improving grain yield and nodulation over that of the non-inoculated control (Hungria et al., 2013). A recent study reported that a composite inoculum of *Pseudomonas chlororaphis* H1 and *Bacillus altitudinis* Y1 could remarkably enhance soybean plant growth, yield performance, enrich the beneficial bacterial composition around root and rhizospheric region with a positive effect on soil improvement (Zhang et al., 2023). Thus, our findings are in line with the previous studies in this arena.

According to Vassileva et al. (2020), the bio-inocula of multi-strain microorganisms with different plant-beneficial properties may exert a consistent impact in on plant productivity in field conditions, either due to the complementation effects of plant-favorable functions at the consortium level or because of imminent diversity effects in the plant-associated microbiome (Hassani et al., 2018). In the present work, enhanced vigor and performance of soybean plant, might have occurred in the consortium-treated condition, due to the composite PGP mechanisms exerted by the three potent PGPB isolates, *Bacillus subtilis* strain MMAM, *Bacillus zhanzhouensis* strain MMAM, and *Bacillus cereus* strain MMAM. Additionally, this study indicated that soil amendment with vermicompost might have an added advantage for plant growth promotion, both in consortium-treated and untreated soil conditions over to that of the only bacterial inoculant treated condition. Vermicompost probably acted as a soil prebiotic to increase the population of resident associative beneficial bacteria and also as a nutrient source for the bacterial strains already existing within the soil–plant system (Arancon et al., 2006; Strachel et al., 2017; Vassileva et al., 2020).

In the recent years, the implications of introduced bioinoculants on soil microbial community composition are extensively investigated. Xing et al. (2022) explored the effect of co-inoculation with three beneficial bacteria (*Bradyrhizobium japonicum* 5,038 (R5038), *Bacillus aryabhattai* MB35-5 (BA) and *Paenibacillus mucilaginosus* 3,016 PM), alone and in combination, on soybean rhizosphere bacterial community composition and on the soil properties. Their findings confirms that several PGPB with multifaceted functions could effectively be used together as composite bacterial inoculants, which coordinately shift the rhizospheric bacterial community structure and improve plant performance. In our study, the analysis of metagenomic data sets of treated and untreated soil, indicated a modulation of soil bacterial community composition following soil augmentation. According to Willis Rarefaction (2019), analysis of the alpha diversity in amplicon sequencing data appears to be a common first approach to measuring differences between environments in terms of microbial ecology to summarize an ecological community structure according to its richness (number of taxonomic groups), evenness (distribution of abundances of the groups) or both. In the present work, the diversity profiles reveals that although the 5th dataset (SVBC) i.e., field soil treated with both vermicompost and bacterial consortia in the presence of soybean plant, had a higher index of Shannon diversity, thus, establishing a higher richness and uniformity in distribution of the total number of genera in the given sample. Our findings are in line with Ansari et al. (2023) who observed a positive correlation between the diversity of soil microbiota and availability of soil nutrients such as, organic carbon, available N and K content, thereby influencing plant growth.

The set of metagenomic analyzes carried out demonstrated that Actinobacteria was found to be the most prevalent phylum in the first two datasets, i.e., untreated field soil and that which was treated with vermicompost in the presence of soybean plant. However, a pronounced shift of phyla was observed in all the amended experimental datasets toward Proteobacteria. This change in abundance could be possibly indicative of the fact that the beneficial Proteobacteria have been shown to exhibit multifaceted roles contributing to plant growth and development such as promoting nutrient balance and acquisition via nitrogen fixation (Mirza et al., 2001; Miliute et al., 2015). The sustained presence of Actinobacteria as one of the common abundant phyla suggests their involvement in nutrient cycling, improvement in soil quality, and enhancing crop yield along with maintenance of plant health thus, being a reliable contender as a biofertilizer alternative to conventional inorganic supplements in agricultural (Boubekri et al., 2022). Various levels of ubiquity were explored as well, regarding the diversity profiles of the bacterial genera across all the datasets including common and unique genera. Some of the rhizobial and PGP bacterial assemblages found to be common among all the treated datasets were *Rhizobium*, *Bradyrhizobium*, *Mesorhizobium*, *Microbacterium*, *Paenibacillus*, *Bacillus*, and *Pseudomonas*, as mentioned in the results section. In soybean plants, acquisition of phosphorus has been specifically evidenced whereby, the inorganic phosphate remobilization via *Rhizobium* is postulated to be mechanistically driven by rhizospheric acidification which is enhanced in the case of modulation between the plant-microbe holobiome (Qin et al., 2011). Furthermore, rhizobial strains have been shown to secrete and/or produce 1-aminocyclopropane 1-carboxylate (ACC) deaminase, siderophores, and extracellular polysaccharide for combating osmotic and heavy metal stresses thus, cumulatively boosting soybean seed germination under drought conditions (Igiehon et al., 2019). Advantages of inoculation of soybean plants with selected strains of *Bradyrhizobium japonicum* exposed to salt stress in greenhouse conditions have been reported to restrict mineral nutrient uptake along with amplified antioxidant activity and production of glutathione reductase, ascorbate peroxidase, and malondialdehyde along with other protective osmolytes thereby, ameliorating the hypersaline microenvironment which otherwise limits the nodulation potential, yield, plant growth and rate of photosynthesis in soybean plants (Han and Lee, 2005). Agricultural sustainability stems majorly from biological nitrogen fixation (BNF), 45% of which is exploited in current agricultural practices. Furthermore, 80% of BNF are contributed by leguminous plant-microbe associations between *Rhizobium*, *Bradyrhizobium*, *Sinorhizobium*, *Azorhizobium*, *Mesorhizobium*, and *Allorhizobium* and their abundances are dictated by ecological, edaphic, genetic and agronomic parameters (Sindhu et al., 2019). Literature sources also reveal that selected strains of *Pseudomonas* have been identified which can substantiate the productivity of the soybean-wheat cropping system in regions of central India with an enhanced content of clayey minerals in the soil, whereby they were found to be boosting soil enzyme activities, total system productivity and nutrient uptake in field trial (Sharma, 2011). Moreover, most pseudomonads have been found to produce phytohormones like IAA along with secondary metabolites including antibiotics with antifungal activity. An interesting study investigated the synergistic effect of inoculating selected strains of *Pseudomonas aeruginosa* with *Bradyrhizobium japonicum* for their potential implication as a biofertilizer consortium for soybean. Compatible strains revealed elevated solubilization of inorganic phosphate and production of IAA, ACC deaminase, and biofilm biosynthesis along with improved grain yield, symbiotic and soil quality parameters compared to independent inoculation with single strains (Kumawat et al., 2019). A specific strain of *Bacillus aryabhattai* has been evidenced to significantly improve the growth of soybean via the synthesis of substantial amounts of abscissic acid, IAA, cytokinins, and GA with subsequent induction of heat stress tolerance (Park et al., 2017). The aerobic endospore-forming bacteria belonging to the genera of *Bacillus* and *Paenibacillus* have been reportedly involved in atmospheric nitrogen fixation, phosphate solubilization, biofilm formation, and production of microbicidal metabolites. They have been evidenced to be mobilizing host plant nutrition thereby supporting their growth, along with antagonizing pathogenic infestations of insect pests, bacteria, fungi, and nematodes by modulation of host defense cascades and triggering induced systemic resistance (ISR) thus making them suitable contenders for application in sustainable agricultural practices (Govindasamy et al., 2010).

During the investigation, a large number of biological pathways were also predicted, which encompassed both homeotic and response pathways. The common metabolic pathways that were found to be of highest prevalence across the soil samples under differential experimental parameters under study were tryptophan metabolism and terpenoid backbone synthesis for the untreated field soil; starch and sucrose metabolism and quorum sensing for field soil in presence of soybean plant; valine, leucine and isoleucine degradation for field soil treated with vermicompost in presence of soybean plant, and all the enriched metabolic cascades were found to be uniformly active in cases of field soil treated with a selected bacterial consortium or with both the consortia and vermicompost in presence of soybean plant. Among the five datasets, the combined treatment of vermicompost with the selected bacterial consortium exhibited a significantly higher magnitude of activation of the most prevalent functional pathways as compared to the remaining datasets in the presented heat map. The selectively enriched pathways of terpenoid backbone synthesis in almost all the datasets can be correlated with existing literature sources which substantiate this functionality in *Salvia miltiorrhiza* seeds from seven different geographic origins whereby, it has shown to provide important precursors for terpenoid biosynthesis thus, indicating a significant level of secondary metabolism for enhancing biotic and abiotic stress resistance (Chen et al., 2018). A significant down-regulation of the sucrose and starch metabolism pathways can be noted for the untreated field soil in the presence of the soybean plant which might be suggestive of the adaptive trait of specialized and dynamic carbon utilization from sources like α-pinene, naphthalene secreted in the root exudates as a part of the unique microenvironment utilized by bacteria like *Pseudomonas*, *Burkholderia*, *Mycobacterium*, *Streptomyces*, *Sphingomonas*, *Pseudomonas*, *Ralstonia*, etc. in the rhizospheric bacterial consortium in soybean thus, leading to a decrease in common carbon metabolism pathways (Liu et al., 2019). Glutathione up-regulation was seen to be considerably activated in most of the soil samples which in synergy with functionalities like geraniol disintegration, limonene, naphthalene, and pinene degradation have potential implications in bioremediation of xenobiotic contamination (Liu et al., 2019).

The soil microbial network analysis of common genera between the 5 experimental conditions exhibits a complex network that represents the interactions that take place between the field soil bacterial assemblages. Here, we found two patterns of interactions, one in which both microbes appear to be communicating with each other. The bidirectional phenomenon is represented by blue lines comprising of members such as *Pseudomonas*, *Solirubrobacter*, *Phenylobacterium*, etc. The second pattern is unidirectional contact which is being mediated by any one member toward the other. This is represented by red lines, exhibited by *Streptomyces*, *Rhodococcus*, *Mycobacterium*, *Paenibacillus*, etc. The edges in between the nodes of the network indicating that some genera such as, *Bacillus*, *Paenibacillus*, *Pseudomonas*, *Streptomyces*, *Nitrospira* are (potentially) co-contributing to one or more specific functions. These findings are supported by the report of Ma et al. (2018), where a significant over-representation of several bacterial classes and genera were observed to be involved in symbiotic N-fixation, plant health promotion, biol-control and soil catalase activity promotion, following bacterial inoculation treatment. Furthermore, a decrease in some taxa with negative impacts on soil quality, was noticed in this study (Ma et al., 2018). The analysis of soil bacterial community also revealed that, application of the microbial consortium resulted in an elevated crosstalk among the microbial members of the niche. Along with an increase in crosstalk, elevated expression of metabolic pathways was also observed, indicating a modulation of resident bacterial assemblage at the community level toward the improvement in soil biological health.

Finally, the results of the current study indicated that the application of the composite inoculant of residual PGPB strains to the soil, in combination with vermicompost, might have enriched the agriculturally beneficial soil microbial assemblages already present in the microbiome and the resultant effects have been reflected in plant growth promotion. This unique soil augmentation strategy has multi-pronged beneficial aspects. Implementation of this technology can effectively enhance vegetative and reproductive performance of plants targeting to increase agricultural productivity. Post-amendment increased abundance of plant beneficial microbial assemblages resulted an enrichment of soil microbial flora leading to an improvement in soil biological health. The rejuvenated patched of over-used land can be used by the small and marginal farmers for cultivation of resilient, as well as profitable crops. In the long run, it can lead to a societal benefit to improve the economic status of the poor farmers. In ecological aspect, popularization of this technology will promote sustainable agriculture. The limitation of the present work is evaluation of the novel strategy at pot trial condition. Extensive field trials are needed to ascertain its implications at field condition usually, some laboratory-tested products fail to exert promising results under field trial conditions. Soybean was used as a model crop in our study, various other cropping systems should also be explored to validate the growth enhancing and stress-relieving behavior of the tested PGPR consortium. Major challenges of this study are: availability of the formulation to the farmers; generation of public awareness and arrangement of proper training facility at the rural perspective for popularizing this novel technology.

## 5 Conclusion

As the world grapples with burgeoning populations and the concomitant challenges of ensuring food security, microbial innovations could be the linchpin. The current work reported that the novel multi-strain inoculant three native Bacillus spp. could remarkably enhance soybean plant growth, yield performance and simultaneously, enrich the resident functional bacterial assemblage in soil. Furthermore, the upgraded reclaimed soil can be successfully used for growing the ‘golden bean’, soybean which is still under-utilized in the state of West Bengal, India, thus helping to improve socio-economic status of the marginal and landless farmers of this region. There is still a scarcity of microbial inoculants-based good products in the market. In this context, the utilization of native BSM to enhance plant productivity in over-exploited nutrient-depleted arable soil through modulation resident microbiome, can emerge as a promising strategy for futuristic agriculture transforming barren patches to fertile expanses, and reducing the environmental footprint of traditional cultivation practices. By harnessing “Emerging Trends and Advances in the Socioe-conomic Applications of Beneficial Microbes,” we are not just looking at scientific advancements but a redefinition of socio-economic paradigms.

## Data availability statement

The datasets presented in this study can be found in online repositories. The names of the repository/repositories and accession number(s) can be found in the article/Supplementary material.

## Author contributions

MM: Data curation, Investigation, Writing – original draft, Conceptualization, Formal analysis, Methodology, Resources, Software, Validation, Writing – review & editing. AM: Formal analysis, Methodology, Software, Validation, Writing – review & editing. SG: Methodology, Methodology, Validation, Writing – review & editing, Data curation. AC: Methodology, Software, Formal analysis, Investigation, Writing – review & editing. SR: Formal analysis, Methodology, Software, Writing – review & editing. SC: Supervision, Validation, Writing – review & editing. VS: Validation, Funding acquisition, Project administration, Resources, Writing – review & editing. VK: Funding acquisition, Project administration, Resources, Validation, Writing – review & editing. AS: Formal analysis, Methodology, Software, Writing – review & editing. FE-D: Formal analysis, Funding acquisition, Methodology, Software, Writing – review & editing. MA: Formal analysis, Funding acquisition, Resources, Software, Writing – review & editing. AŞ: Formal analysis, Resources, Software, Validation, Writing – review & editing. BD: Data curation, Investigation, Project administration, Writing – review & editing. AKM: Conceptualization, Investigation, Project administration, Supervision, Validation, Writing – review & editing.

## Footnotes

^1^https://agritech.tnau.ac.in/agriculture/agri_soil_sampling.html/TNAU-2013
