# Supplementary material for: Marvels of Bacilli in soil amendment for plant-growth promotion toward sustainable development having futuristic socio-economic implications
Source: Front Microbiol. 2023 Dec 7;14:1293302. doi: 10.3389/fmicb.2023.1293302 (PMC10752760; doi:10.3389/fmicb.2023.1293302)
Supplement: Supplementary file 1 [file Table_1.docx]

**Supplementary Tables**

| **Supplementary Table 1:** Tests of Normality | | | | | | |
| --- | --- | --- | --- | --- | --- | --- |
|  | **Kolmogorov-Smirnov^a^** | | | **Shapiro-Wilk** | | |
|  | **Statistic** | **df** | **Sig.** | **Statistic** | **df** | **Sig.** |
| Total_leaves_SU | .138 | 12 | **.200^*^** | .975 | 12 | **.958** |
| Total_chlorophyll_SU | .202 | 12 | **.192** | .882 | 12 | **.093** |
| root_nod_SU | .226 | 12 | **.091** | .859 | 12 | **.048** |
| pods_plant_SU | .230 | 12 | **.080** | .900 | 12 | **.160** |
| DRY_WT_SU | .287 | 12 | **.007** | .783 | 12 | **.006** |
| pr_SU | .159 | 12 | **.200^*^** | .928 | 12 | **.355** |
| leaves_SVBC | .180 | 12 | **.200^*^** | .957 | 12 | **.748** |
| chl_SVBC | .197 | 12 | **.200^*^** | .911 | 12 | **.219** |
| root_nod_SVBC | .145 | 12 | **.200^*^** | .948 | 12 | **.615** |
| pods_plant_SVBC | .205 | 12 | **.176** | .890 | 12 | **.118** |
| dry_wt_100_seeds_SVBC | .156 | 12 | **.200^*^** | .965 | 12 | **.849** |
| Protein_content_seeds_SVBC | .145 | 12 | **.200^*^** | .932 | 12 | **.400** |
| a. Lilliefors Significance Correction | | |  |  |  |  |
| *. This is a lower bound of the true significance. | | | |  |  |  |

SU. Experimental set-up-1. Untreated field soil; SVBC. Experimental set-up-4. Field soil treated with vermicompost and bacterial consortium.

**Supplementary Table 2:** Paired sample t-Test

| **Pair No.** | **Variables** | **t-value** | **df** | **P-value** |
| --- | --- | --- | --- | --- |
| 1 | Leaves_SU_SVBC | 27.567 | 11 | .000 |
| 2 | Chl (a+b)_SU_SVBC | -90.312 | 11 | .000 |
| 3 | Root_nodule_SU_SBVC | 34.413 | 11 | .000 |
| 4 | pods_plant_SU_SVBC | 86.216 | 11 | .000 |
| 5 | Dry_wt_100_seeds_SU_SVBC | 49.542 | 11 | .000 |
| 6 | Protein_content_seeds_SU_SVBC | 30.488 | 11 | .000 |

**Supplementary Table 3:** QC Parameters of the given Paired-End Miseq Illumina Sequences

| **DATASET** | **Sample Code** | **Total Reads** | **QC**  **Passed** | **Read Length** | **Total GC**  **Content (in %)** | **PHRED Score** |
| --- | --- | --- | --- | --- | --- | --- |
| 1 | S | 651486 | 616222 | 150 | 57 | 38 |
| 2 | SU | 631473 | 578291 | 150 | 57 | 38 |
| 3 | SV | 105984 | 105984 | 602 | 57 | 22 |
| 4 | SBC | 153600 | 153600 | 602 | 57 | 22 |
| 5 | SVBC | 90810 | 90810 | 602 | 57 | 22 |

**Supplementary Fig 5.** Bacterial abundance in SVBC (Field soil treated with vercompost +bacterial concortia+soybean plants) condition. a. Krona chart representation of Phyla level abundance of prevalent bacterial assemblage, b. Krona chart representation of Genera level abundance of prevalent bacterial assemblage , c. Pie chart representing the top 10 scoring genera.

**c**
